# Synchrotron mechanism of X-ray and gamma-ray emissions in lightning and spark discharges

**DOI:** 10.1038/s41598-021-99336-3

**Published:** 2021-10-06

**Authors:** N. I. Petrov

**Affiliations:** Scientific and Technological Centre of Unique Instrumentation of the Russian Academy of Sciences, 15 Butlerova str., Moscow, Russia 117342

**Keywords:** Plasma-based accelerators, Atmospheric dynamics

## Abstract

X-ray and γ-ray emissions observed in lightning and long sparks are usually connected with the bremsstrahlung of high-energy runaway electrons. Here, an alternative physical mechanism for producing X-ray and gamma-ray emissions caused by the polarization current and associated electromagnetic field moving with relativistic velocity along a curved discharge channel has been proposed. The existence of fast electromagnetic surface waves propagating along the lightning discharge channel at a speed close to the speed of light in vacuum is shown. The possibility of the production of microwave, X-ray and gamma-ray emissions by a polarization current pulse moving along a curved path via synchrotron radiation mechanism is pointed out. The existence of long tails in the power spectrum is shown, which explains observations of photon energies in the range of 10–100 MeV in the terrestrial gamma-ray flashes, as well as measured power spectrum of laboratory spark discharge.

## Introduction

Lightning discharges are the most common source of powerful electromagnetic fields of natural origin. Lightning radiation covers almost the entire wavelength range, from a few hertz to ultraviolet. In^1^, it was suggested that strong electric fields in thunderclouds can cause nuclear reactions. Recently, the first observational evidence of photonuclear reactions triggered by lightning discharge was reported^2^. In recent decades, a phenomenon of terrestrial gamma-ray flashes (TGF) generated during thunderstorms was discovered^3^. X-ray and gamma-ray flashes from natural^4,5^ and rocket-triggered^6^ lightning, as well as from laboratory spark discharge were observed^7^. TGF photons are usually assumed to be produced via the bremsstrahlung of runaway electrons accelerated by strong electric fields in the atmosphere^8^. Currently, two models of electron acceleration and multiplication based on the relativistic runaway electron avalanche (RREA) mechanism^9,10^ and the lightning leader model^11,12^ have been proposed. These models explain the multiplication of energetic electrons and the subsequent production of bremsstrahlung photons.

However, some basic properties, such as the radiation spectrum (in the region above 10 MeV), the cut-off energy, and the polarization and beaming characteristics of the radiation still need to be explained. Clarifying the relationship between the parameters of TGF and the characteristics of lightning discharge channel is also important.

From ground-based observations, it follows that TGFs are associated with cloud-to-ground discharges of negative polarity. Measurements show that X-rays from natural lightning and intense bursts of gamma-ray radiation with energies up to 10 MeV are correlated with negative leader stepping^9,10^. The detection of TGF emission with photon energies in the 10–100 MeV range was reported in^13^. It was shown that the detected power-law radiation in the range from 10 to 100 MeV is difficult to explain using RREA models^9–12^.

Recent observations have shown that gamma radiation correlates with radio-frequency radiation and is generated at the last stage of lightning leader channel development prior to the lightning return stroke^14^. The bursts of 30–80 MHz radiation because of leader stepping were observed in^15^. It was shown in^16^ that TGF is produced in the initial stage of a lightning flash just before the initiation of the current pulse. Observations of TGFs which occur at the onset of UV and optical emissions also point to the importance of lightning leaders^16–18^.

In this paper, the synchrotron mechanism of production of X-ray and *γ*-ray emissions by polarization current and associated surface electromagnetic wave propagating along the ionized lightning channel with relativistic velocities is proposed. The influence of the conductivity and the radius of the lightning channel on the propagation velocity of electromagnetic waves, taking into account the absorption, has been investigated. The possibility of the production of X-ray and gamma-ray emissions from a polarization current pulse moving along a curved path during the lightning leader steps formation and last stage of lightning leader channel (final jump phase—the very beginning of the first and subsequent return stroke stages) is pointed out.

## Results

### Physical model

The streamer-leader process underlies the development of lightning and spark discharges in the atmosphere^10^. The embedded charges distributed within the leader channel are neutralized during the leader step processes or during the lightning return stroke. It is established that the front of the neutralization process moves along the channel at a speed of the order of the speed of light^10^. High frequency electromagnetic wave modes are excited in the lightning discharge channels during the leader step processes or during grounding. It is well known that the surface electromagnetic waves can propagate along the conducting wire^19^. Here, we show that fast surface electromagnetic waves with the velocities close to the speed of light in vacuum can propagate along the ionized channel of the lightning leader over a limited distance.

The electromagnetic field behaviour in a lightning channel is described by the dispersion equation which is followed from the Maxwell equations. Dispersion equation for the surface electromagnetic waves is followed from the boundary condition of continuity of the tangential components of the field at $r = r_{0}$:

1 $$\frac{{\varepsilon_{p} }}{\eta a}\frac{{I_{0}^{{\prime }} \left( {\eta a} \right)}}{{I_{0} \left( {\eta a} \right)}} = \frac{1}{{\eta_{0} a}}\frac{{K_{0}^{{\prime }} \left( {\eta_{0} a} \right)}}{{K_{0} \left( {\eta_{0} a} \right)}},$$

where $I_{0}$ and $K_{0}$ are the modified Bessel functions of the first and second kind, $I_{0}^{{\prime }}$ and $K_{0}^{{\prime }}$ are the derivatives of the Bessel functions, $\eta^{2} = k_{0}^{2} \varepsilon_{p} - \beta^{2}$, $\eta_{0}^{2} = k_{0}^{2} - \beta^{2}$, $k_{0} = \frac{\omega }{c}$ is the wavenumber in free space, $\beta$ is the longitudinal component of the wavenumber, $r_{0}$ is the channel radius, $\varepsilon_{p} = \varepsilon^{{\prime }} + i\frac{\sigma }{{\omega \varepsilon_{0} }}$ is the complex dielectric constant, where $\sigma = \frac{1}{{R_{l} \pi r_{0}^{2} }}$ is the electric conductivity, $R_{l}$ is the resistance per unit length, and $\varepsilon_{0}$ is the dielectric constant of free space.

Phase and group velocities of the surface wave can be determined from the dispersion Eq. (1).

The wavevector $\beta = \beta^{{\prime }} + i\beta^{{\prime \prime }}$ is a complex value. The real part $\beta^{{\prime }}$ defines the phase velocity $V_{ph} = \frac{\omega }{{\beta^{\prime}}}$ of the wave, and the group velocity is determined by $V_{g} = \frac{d\omega }{{d\beta^{\prime}}}$. The imaginary part $\beta^{{\prime \prime }}$ defines the attenuation length $z_{0} = \frac{1}{{\beta^{{\prime \prime }} }}$ of the surface wave propagating along the discharge channel.

**Figure 1:**
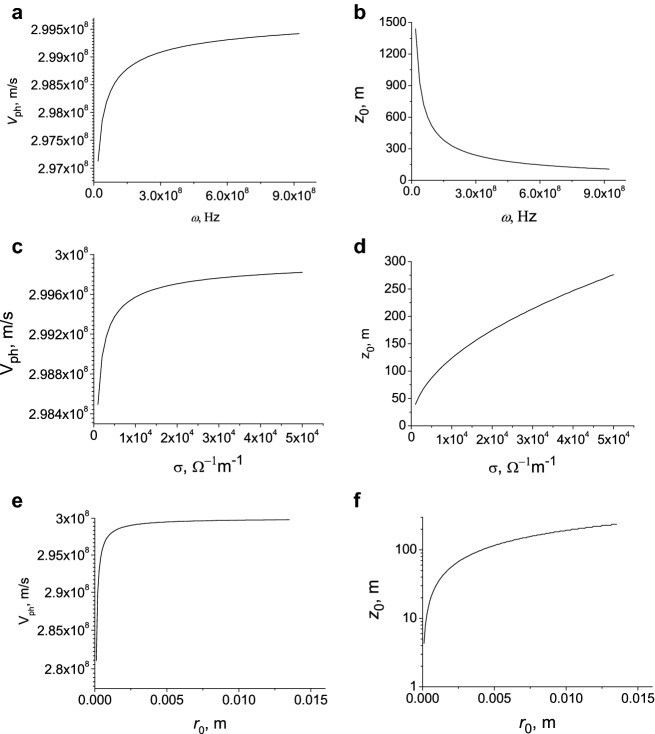
Velocity (panels a, c and e) and attenuation length (panels b, d and f) as function of frequency (**a**, **b**), conductivity (**c**, **d**) and channel radius (**e**, **f**). (**a**, **b**)-σ = 10^4^ $\Omega^{ - 1} m^{ - 1}$, $r_{0}$ = 0.01 m; (**c**, **d**)-$\omega = 2\pi f = 10^{9}$ Hz, $r_{0}$ = 0.01 m; (**e**, **f**)-$\omega = 2\pi f = 3 \cdot 10^{10}$ Hz, σ = 10^5^ $\Omega^{ - 1} m^{ - 1}$.

In Fig. 1 the velocity and the attenuation length of the surface wave as a function of the frequency, conductivity and radius of the discharge channel are presented.

It follows from the simulation that the velocity increases with the frequency. The propagation distance of the surface wave decreases when the frequency increases (Figs. 1a,b). Only the components of the current pulse with frequencies of the order of MHz and lower reach high altitudes of the order of a kilometer or more. For a given frequency the velocity is higher for the higher electric conductivity of a channel and the propagation distance increases with the conductivity (Figs. 1c,d). The velocity and propagation distance of the surface waves increase with the radius of a discharge channel (Figs. 1e,f). Thus, the speed of surface waves increases with frequency, as well as with the conductivity and radius of a discharge channel (Fig. 1). These waves are attenuated during propagation along the channel because of the skin effect. Dissipation increases with frequency because of the skin effect. The propagation distance of surface waves decreases with frequency because of dissipation (Fig. 1b). However, this distance increases with the conductivity and radius of a discharge channel (Fig. 1d,e). It follows that ultra-relativistic velocities of the order of the speed of light in vacuum are achieved for high-frequency waves at the conductivities and radii of the discharge channel characteristic of lightning.

At present, there is a sufficient amount of measured data on the parameters of lightning and spark discharge in the laboratory. In^20^ the diameter of the lightning stroke was measured by allowing lightning discharges to pass through fiberglass screen. The diameters of the holes produced in fiberglass screen were varied from 2 mm to 3.5 cm. It was shown in^21^ that the channel diameter determined from the 224 images obtained with a high-speed framing camera has a mean value of 6.5 cm.

Note that the surface plasmon wave causes an additional ionization resulting in generation of the higher plasma density. It was shown that the electron density of the lightning stepped leader is of the order of 10^24^ m^−3^ ^22^. The measurements show that the resistances per unit length of the lightning channel are of the order of 10^–2^–10^–1 ^Ω/m and the internal electric field strengths are of the order of 10^3^ V/m^23^. For the resistance per unit length *R*_*l*_ = 10^–2^ Ω/m and the channel radius $r_{0}$ = 6 mm we have the conductivity $\sigma = \frac{1}{{R_{l} \pi r_{0}^{2} }} \approx \frac{1}{{10^{ - 2} \cdot 10^{ - 4} }} \sim 10^{6}$ Ω^−1^ m^−1^. The same order of conductivity follows from the Drude model for the electron density of the lightning leader *n* = 10^24^ m^-3^. In the laboratory spark discharges the conductivity has lower values: *R*_*l*_ ~ 10 Ω/m^24,25^.

The calculations show that a fast surface electromagnetic wave of high frequency propagates on the surface of the plasma channel at a speed close to the speed of light in vacuum. Surface plasmon waves, which are the combination of the polarization density (electric dipoles) wave and the electromagnetic wave, propagate along the lightning leader channel. These high frequency guided waves are generated during the lightning leader steps formation and during grounding (final jump phase—the very beginning of the return stroke), as well as during the formation of the subsequent return strokes.

Excitation of high-frequency electromagnetic waves in lightning and spark discharges is confirmed by the measurements^26,27^. The surface plasmon waves can be interpreted as ionizing waves of the potential gradient^28^. Note that the propagation of fast ionization waves under electrical breakdown conditions were experimentally demonstrated in^29^. It was shown that the shorter the pulse front of the applied voltage and the stronger the pre-ionization of the discharge gap, the greater the speed of the ionization wave starting from the high-voltage electrode.

The measured speeds of the return stroke and illumination pulses are less than the speed of light^30,31^. Optical measurements of return strokes speed are not available during the initial stages of natural lightning return strokes, but it can be evaluated from the measured electric fields and electric field derivatives. It was shown in^32^ that the initial return stroke speed is actually near the speed of light *c* for the bottom 30 m of the triggered lightning channel. The current risetime increases with the height from the ground indicating that high frequency components propagate only for a short distance.

Note that the velocity values are usually underestimated due to the tortuosity of the breakdown channel. The real length of a tortuous and oblique channel is larger due to fractal nature of a lightning channel^33^. This implies that ultra-relativistic velocities for high-frequency surface plasmon waves in a lightning discharge channel are realistic. Calculations show that the velocities of the order of *c*/2 correspond to the moderate values of the conductivity of the lightning channel.

### Radiation of e/m waves

**Figure 2:**
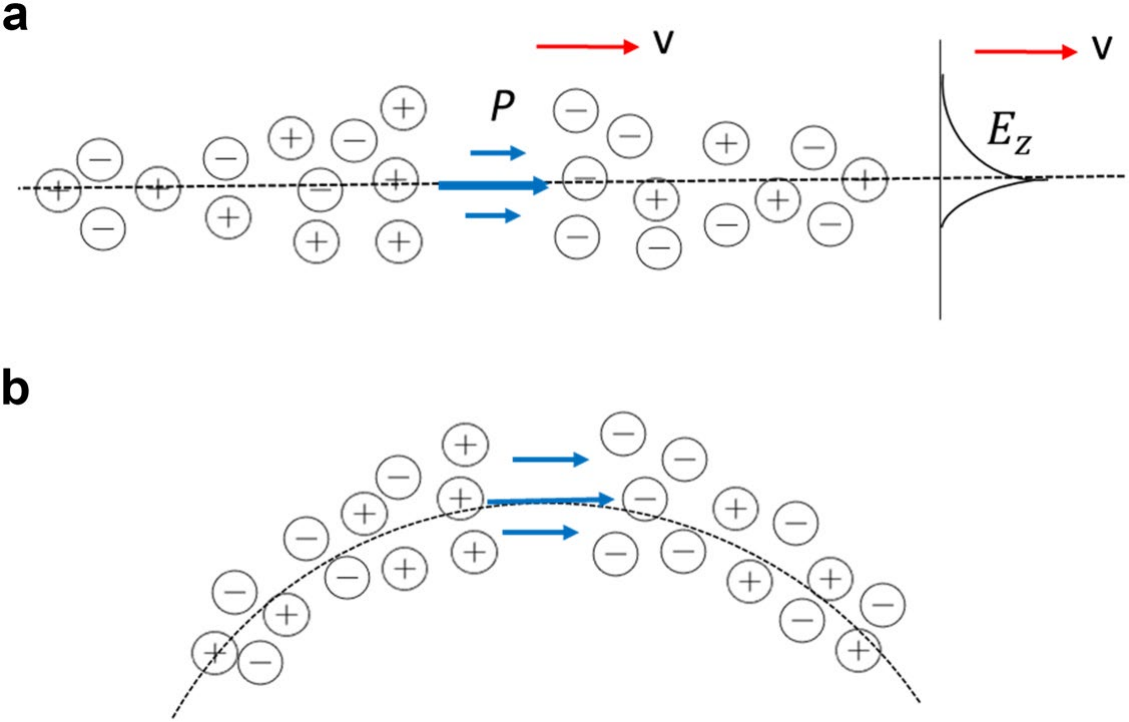
Simplified representation of the motion of a polarized region. The velocity v coincides with the velocity of the surface electromagnetic field. In (**a**) on the right, the exponential dependence of the electromagnetic field intensity on the distance away from the interface is shown; (**b**) the channel curvature introduces centripetal acceleration of the moving polarized region and electrons.

An electromagnetic wave in a plasma channel results in a time-dependent electric dipole (polarization) that generates a polarization current pulse propagating at a relativistic speed. It follows from the laws of electrodynamics that charged particles or dipoles moving with acceleration (or oscillation) should emit electromagnetic radiation. A polarization current pulse moving along a curved (circular) path generates a radiation similar to the synchrotron radiation produced by electrons circulating in a magnetic field. Figure 2 shows a simplified model of induced polarization motion along the surface of a plasma channel. Spatially decaying electric field causes the negative and positive charges to move in opposite directions, i.e., a polarization *P* is induced. The movement of the polarized region with a velocity $v \approx c$ causes the polarization current $j_{pol} = \frac{\partial P}{{\partial t}} = \frac{\partial P}{{\partial z}}\frac{\partial z}{{\partial t}} = \frac{1}{4\pi }\left( {\varepsilon - 1} \right)\varepsilon_{0} \frac{{\partial E_{z} }}{\partial z}v = \frac{{\left( {\varepsilon - 1} \right)}}{\varepsilon }\rho v$, where $\rho = n_{e} - n_{i}$ is the charge density. Here, the relation between electric induction and polarization $\vec{D} = \varepsilon \varepsilon_{0} \vec{E} = \varepsilon_{0} \vec{E} + 4\pi \vec{P}$ and the Poisson equation $\varepsilon_{0} div\varepsilon \vec{E} = 4\pi \rho$ are used for the *z*-components of polarization and the electric field.

**Figure 3:**
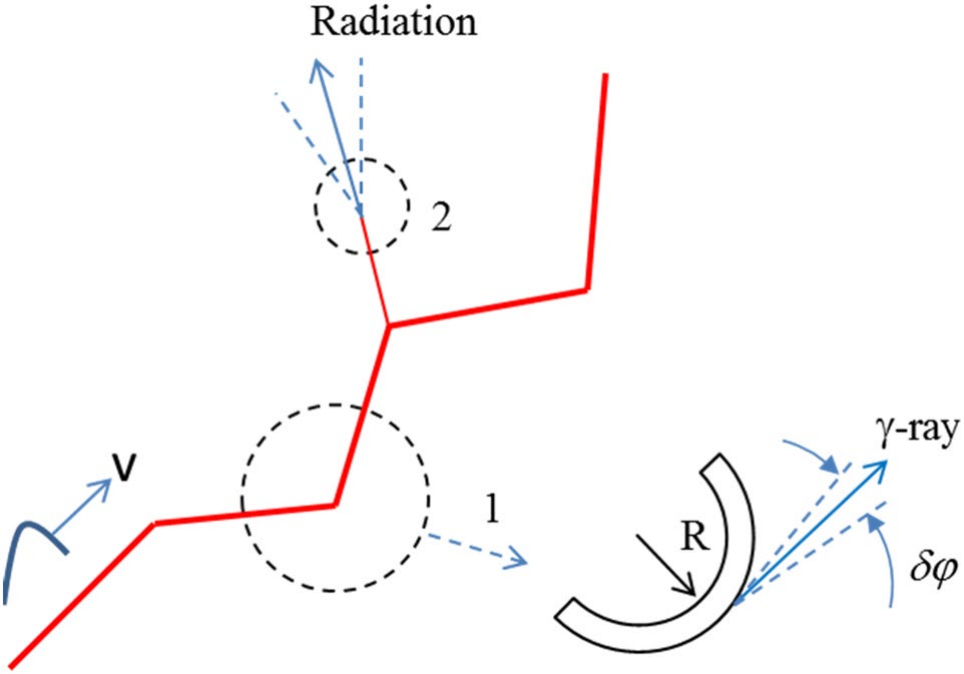
Schematic view of a lightning channel. Sources (places) of radiation: 1–synchrotron radiation; 2–transition radiation.

There are numerous curvatures and irregularities on the tortuous lightning channel boundary (Fig. 3). The curvature of the trajectory introduces centripetal acceleration in the moving polarized region, thereby leading to electromagnetic radiation.

The induced polarization defines the electric dipoles density, so the properties of the radiation are determined by the emission resulting from the motion of a dipole or from a charge *e* that moves with relativistic speed along a circular trajectory. The contribution of ions to the plasma polarization is only a small correction of the order of the ratio of the masses of electrons and ions $m_{e} /M_{i} \sim$ 10^–3^, and it can be neglected. Note that the velocity of the electrons involved in the conduction current is much less than the velocity of the polarization waves. However, the polarization induced by the surface electromagnetic field propagates along the lightning leader channel with an ultra-relativistic velocity at relatively high conductivities of the lightning channel. The polarized region propagating together with the surface electromagnetic field has a negative charge due to the excess of negative charges (electrons) in the discharge channel before the return stroke. Indeed, X-rays were observed only during the formation of the lightning leader steps before the return stroke with the highest peak current.

The electromagnetic radiation generated by charges moving along a curved trajectory (synchrotron radiation) has been well known for a long time^34^.

The power emitted into the *m-*th harmonic is given by^35,36^:

2 $$W\left( m \right) = \frac{{e^{2} cm\alpha }}{{4\pi \varepsilon_{0} R^{2} }}\left[ {2\alpha^{2} J_{2m}^{^{\prime}} \left( {2m\alpha } \right) + \left( {1 - \alpha^{2} } \right)\mathop \smallint \limits_{0}^{2m\alpha } J_{2m} \left( x \right)dx} \right],$$

where $\alpha = \frac{v}{c}$, *m* is the harmonic order, *R* is the curvature radius of the trajectory bend.

In the non-relativistic case, the main contribution to the total power $W = \mathop \sum \limits_{m = 1}^{\infty } W\left( m \right)$ gives the radiation of the first harmonic (*m* = 1, dipole radiation). The total radiation power of a non-relativistic electron is^36^:

3 $$W\left( 1 \right) = \frac{2}{3}\frac{{e^{2} c\alpha^{4} }}{{4\pi \varepsilon_{0} R^{2} }}.$$

In the ultra-relativistic case, the total radiation power is given by^35,36^:

4 $$W = \frac{2}{3}\frac{{e^{2} c}}{{4\pi \varepsilon_{0} }}\frac{{\alpha^{4} \gamma^{4} }}{{R^{2} }},$$

where $\gamma = \left( {1 - \frac{{v^{2} }}{{c^{2} }}} \right)^{{ - \frac{1}{2}}}$ is the relativistic Lorentz factor, $\alpha = \frac{v}{c}$, *e* is the electron charge, v is the velocity of the surface plasmon wave (polarization density), *c* is the speed of light in vacuum, and *R* is the curvature radius of the trajectory bend.

### Radiation power spectrum

Power spectrum is defined by^36^

5 $$\frac{dW}{{dy}} = WF\left( y \right),$$

where $F\left( y \right) = \frac{9\sqrt 3 }{{8\pi }}y\mathop \smallint \limits_{y}^{\infty } K_{\frac{5}{3}} \left( x \right)dx$, $K_{p} \left( x \right)$ is the Macdonald function, $y = \frac{\omega }{{\omega_{c} }}$, $\omega_{c} = \omega_{0} \gamma^{3}$.

The spectral power density of the radiation in the low frequency region ($y \ll 1$) is given by

$$\frac{dW}{{d\omega }} \simeq \frac{{e^{2} \omega_{c} }}{{c\varepsilon_{0} \gamma^{2} }}\left( {\frac{\omega }{{\omega_{c} }}} \right)^{\frac{1}{3}} .$$

In the high-frequency region ($y \gg 1$) the spectral power density has the form:

$$\frac{dW}{{d\omega }} \simeq \frac{{e^{2} \omega_{c} }}{{c\varepsilon_{0} \gamma^{2} }}\left( {\frac{\omega }{{\omega_{c} }}} \right)^{\frac{1}{2}} \exp \left( { - \frac{2}{3}\frac{\omega }{{\omega_{c} }}} \right).$$

**Figure 4:**
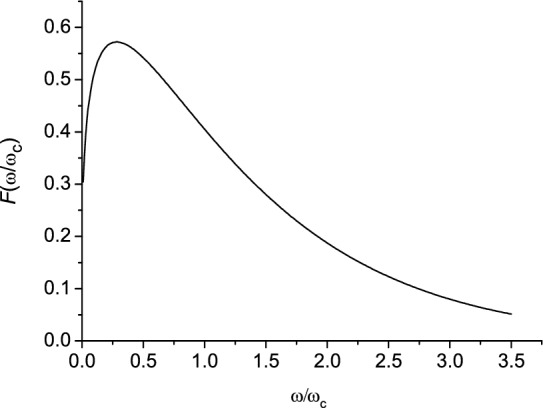
Spectral distribution of the radiation.

In Fig. 4 the spectral distribution of the radiation is presented. The spectral distribution has a maximum near $\omega \approx \frac{{\omega_{c} }}{3}$, where $\omega_{c} = \left( \frac{3}{2} \right)\omega_{0} \gamma^{3}$, and the main part of the radiation is concentrated in this frequency range. Note that the synchrotron radiation spectrum is discrete^34^. However, the radiation spectrum consists of a very large number of closely spaced lines (individual high harmonics are not spectrally resolved), and, consequently, in the ultrarelativistic case of electron motion, synchrotron radiation has an almost continuous spectrum^36^. The unique properties of synchrotron radiation are a sharp angular directivity, strong linear polarization, and a wide spectrum with a maximum in the high frequency region.

The maximum in the spectral power distribution is achieved at the frequency $\omega_{max} = \frac{1}{2}\gamma^{3} \omega_{0}$, where $\omega_{0} = \frac{v}{R}$^36^. The radiation is concentrated mainly in a narrow cone with an axis along the direction of the velocity. The angular width ∆θ in which the main part of the radiation is enclosed is inverse proportional to the relativistic Lorentz factor: $\Delta \theta \sim \frac{1}{\gamma }$. This indicates that the ultra-relativistic velocities of the polarization bunch lead to a very narrow spatial distribution of X-rays and gamma rays.

Strong linear polarization of radiation occurs in the orbital plane. Moreover, the degree of polarization is equal to 3/4^36^. Total powers of σ-and π-polarization components $W_{\sigma }$ and $W_{\pi }$, are equal to $W_{\sigma } = \frac{7}{8}$ and $W_{\pi } = \frac{1}{8}$^36^. In the plane of the orbit of revolution, the radiation is completely linearly polarized, since $W_{\pi } = 0$. Note that polarization can serve as an accurate criterion for testing hypotheses about the nature of radiation. In particular, according to measurements of the polarization of electromagnetic radiation from the Crab Nebula, the synchrotron nature of radiation was established^37^.

The spectral power density (5) can be transformed into an energy distribution of the photon flux, which is the number of photons of energy *E*_*ph*_ emitted per second ($\dot{N} = dN/dt$), into the energy band *dE*_*ph*_:

$$\begin{aligned} & dW = E_{ph} d\dot{N} = E_{ph} \frac{{d\dot{N}}}{{dE_{ph} }}dE_{ph} , \\ & \frac{dW}{{d\omega }} = \hbar E_{ph} \frac{{d\dot{N}}}{{dE_{ph} }}, \\ & \frac{{d\dot{N}}}{{dE_{ph} }} = \frac{1}{{\hbar E_{ph} }}\frac{dW}{{d\omega }} = \frac{W}{{E_{ph} E_{c} }}F\left( {\frac{\omega }{{\omega_{c} }}} \right),E_{c} = \hbar \omega_{c} . \\ \end{aligned}$$

**Figure 5:**
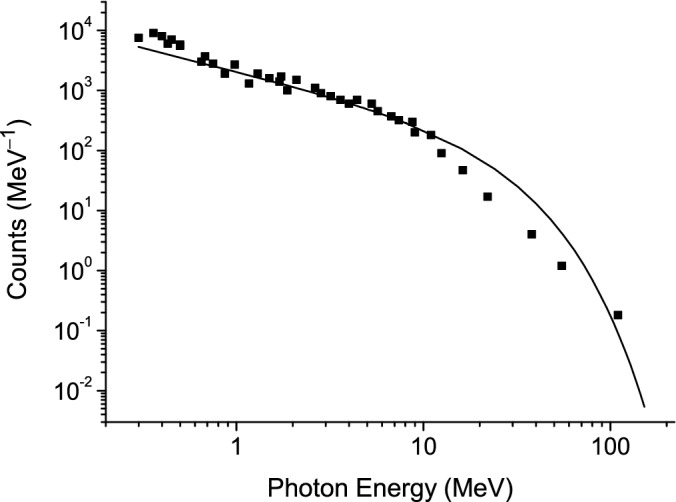
The normalized photon spectrum (solid curve) and measured spectrum from AGILE^13^. *E*_c_ = 16 MeV.

In Fig. 5 the calculated and measured photon counts $d\dot{N}$ per energy band *dE*_*ph*_ are presented.

It is seen that there is not exponential cutoff near 10 MeV in contrast to RREA models.

Our model is also consistent with the measured spectrum from laboratory spark discharge^7^. Note, that nanosecond-fast X-ray bursts in laboratory discharges are accompanied by high-frequency oscillations of the pre-breakdown current^7^.

**Figure 6:**
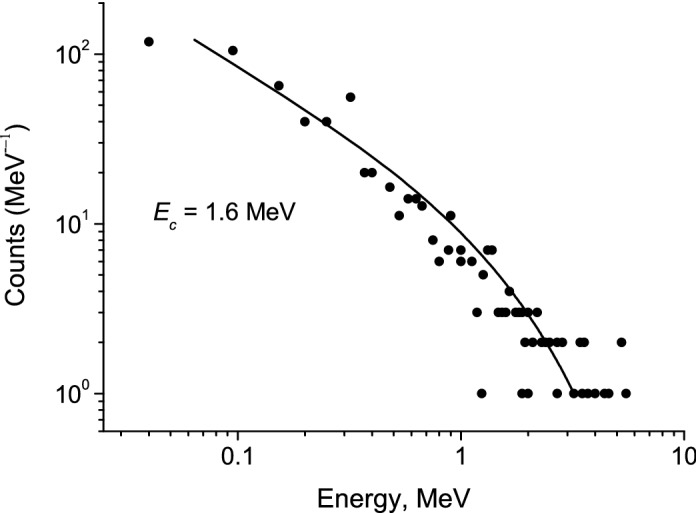
The normalized photon spectrum (solid line) and measured spectrum from laboratory spark discharge^7^. *E*_c_ = 1.6 MeV.

In Fig. 6 the calculated and measured photon counts are presented. A power-law spectrum is seen for energies less than 1 MeV, while the exponential decrease is observed at high energies.

## Discussion

It follows from the simulation that the conductivity and radius of the discharge channel are the main critical parameters to initiate the X-ray and gamma-ray emissions. High conductivity can be created by the return stroke current, so the gamma-ray emission is produced by the pulses moving along a channel after the start of the return stroke. It was shown in^4^ that the gamma-ray flashes with the highest energy exceeding 20 MeV occurred well after the start of the return stroke. This is consistent with our result that the gamma-ray flash with harder energy spectrum is associated with the higher conductivity and larger radius of a lightning channel. TGF flashes from the leader steps should have softer energy spectrum because of the lower conductivity and smaller radius of a leader channel.

It was shown recently, that TGFs are produced during strong initial breakdown pulses (IBPs) in the beginning stages of negative-polarity breakdown^39,40^. Similar effect was shown in^41^, where X‐rays were only observed during the leader before the return stroke with the highest peak current.

Depending on the conductivity and radius of a discharge channel, the energy spectrum of the radiation extends from the radio-frequency to X-ray and gamma-ray. This indicates that the same source regions are responsible for these radiations. Indeed, the correlation of X-ray emission with radio wave radiation is recorded in measurements in^15,42^.

Recently radio-frequency radiation (30–80 MHz) which correlates with the X-ray and gamma-ray radiations was observed at each step of a negative lightning leader^15^. It was shown that the vhf emission is concentrated near the tip of the leader, as well as along the body of the step.

We assume that high intensity radio-frequency pulses from a lightning discharge are emitted by a coherent mechanism, and X-rays and gamma rays can be interpreted using the mechanism of incoherent synchrotron radiation.

In contrast to TGF models based on the RREA process^4,9–12^, which produce a typical electron energy spectrum close to exponential, our model gives a long tails in the photon power spectrum.

It follows from our model that the X-ray and gamma-ray radiations are concentrated in a narrow cone. Spatial location of the source region within discharge channel and the radiation pattern (orientation) depend also on the tortuosity and branches of the channel. This will cause the strong dependence of the detected radiation power on the direction of the detector. It was shown in^41^ that the orientation of the descending leader plays an important role in the detection of X‐rays. It was also pointed out in^7^, that the X-ray bursts in a laboratory spark discharge have a finite opening cone.

Note that the proposed synchrotron radiation mechanism, unlike the existing models, does not require a large-scale region of a high-intensity electric field to accelerate the electrons and seed particles necessary for the RREA processes.

## Conclusions

To conclude, the theoretical model of X-ray and gamma-ray emissions, as well as radio-frequency radiation produced by Lightning discharge via synchrotron and transition radiation mechanisms is consistent with the observational data. The proposed model is characterized by a wide spectral range on the scale of electromagnetic waves and acute collimation, which provides high source brightness, high power, and strong linear polarization of the radiation. The source of synchrotron radiation of lightning is the polarization density distribution (the pulse of the polarization current) and associated surface electromagnetic wave, moving along a curved and irregular ionized channel with a relativistic velocity. The existence of long tails in the power spectrum is shown, which explains observations of photon energies in the range of 10–100 MeV in the TGF. Polarization can serve as an accurate criterion for testing hypotheses about the synchrotron nature of lightning high-energy radiation. Therefore, observations of the polarization and beaming properties are necessary to confirm the synchrotron nature of the X-ray and gamma-ray radiation produced by a lightning discharge.

## Methods

For the cylindrical structure of plasma channel the guided modes may be determined from the Helmholtz equations for the longitudinal field component *E*_z_:

6 $$\begin{aligned} & \left[ {\nabla_{ \bot }^{2} + \left( {k_{0}^{2} \varepsilon_{p} - \beta^{2} } \right)} \right]E_{z} = 0,\;0 < r < r_{0} \\ & \left[ {\nabla_{ \bot }^{2} + \left( {k_{0}^{2} - \beta^{2} } \right)} \right]E_{z} = 0,\;r > r_{0} , \\ \end{aligned}$$

where $\nabla_{ \bot }^{2} = \frac{1}{r}\frac{\partial }{\partial r}\left( {r\frac{\partial }{\partial r}} \right) + \frac{1}{{r^{2} }}\frac{{\partial^{2} }}{{\partial \varphi^{2} }}$, $k_{0} = \frac{\omega }{c}$ is the wavenumber in free space, $\beta$ is the longitudinal component of the wavenumber, $r_{0}$ is the channel radius, $\varepsilon_{p} = \varepsilon^{\prime} + i\frac{\sigma }{{\omega \varepsilon_{0} }}$ is the complex dielectric constant, where $\sigma = \frac{1}{{R_{l} \pi r_{0}^{2} }}$ is the electric conductivity, $R_{l}$ is the resistance per unit length, and $\varepsilon_{0}$ is the dielectric constant of free space.

Solutions of the Eq. (6) are the Bessel functions:

7 $$E_{z} = \left\{ {\begin{array}{*{20}l} {A_{1} I_{0} \left( {\eta r} \right),} \hfill & {r \le r_{0} } \hfill \\ {A_{2} K_{0} \left( {\eta_{0} r} \right),} \hfill & {r \ge r_{0} } \hfill \\ \end{array} } \right.,$$

where *A*_1_ and *A*_2_ are the amplitude coefficients, $I_{0}$ and $K_{0}$ are the modified Bessel functions of the first and second kind, $\eta^{2} = \left( {\frac{{\omega^{2} }}{{c^{2} }}} \right)\varepsilon_{p} - \beta^{2}$, $\eta_{0}^{2} = \left( {\frac{{\omega^{2} }}{{c^{2} }}} \right) - \beta^{2}$.

The wave fields (7) are localized near the plasma-air boundary, so the wave is a surface wave propagating along the channel boundary.

### Velocity of surface wave as function of channel conductivity

**Figure 7:**
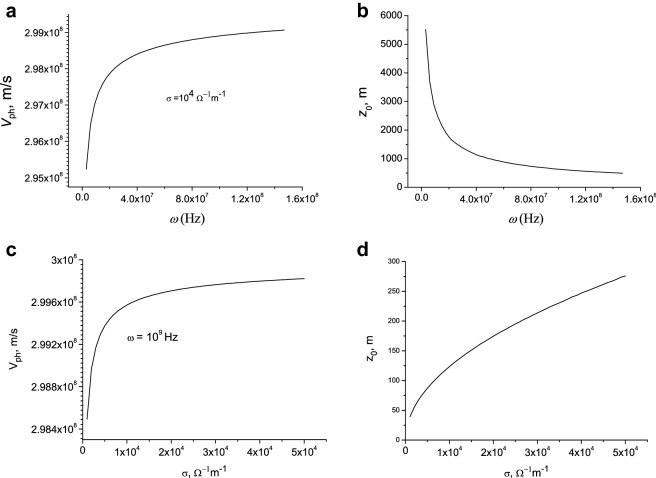
Velocity (panels a and c) and attenuation length (pabels b and d) as function of frequency and conductivity: $r_{0}$ = 0.01 m. (**a**, **b**)-σ = 10^4^ $\Omega^{ - 1} m^{ - 1}$; (**c**, **d**)-$\omega = 2\pi f = 10^{9}$ Hz.

Figure 7 shows the velocity and attenuation length of the surface wave as a function of the frequency and conductivity of the discharge channel at moderate values of conductivity.

It follows from the simulation that the speed is less than the speed of light in a vacuum and their values are in the range of the observational data. This indicates that the measurements were made for lightning discharges with average channel conductivity values.

The velocity of surface waves increases with frequency, as well as with the conductivity and radius of the discharge channel (Fig. 7). These waves are attenuated when propagating through the channel due to the skin effect. The dissipation increases with frequency due to a decrease in the thickness of the skin layer: $\delta = \left( {\frac{{2\varepsilon_{0} c^{2} }}{\omega \sigma }} \right)^{\frac{1}{2}}$. The propagation distance of surface waves decreases with the increase of the frequency due to dissipation (Fig. 7). However, this distance increases with increasing conductivity and the radius of the discharge channel.

It follows from the simulation that phase velocities of the order of the speed of light in vacuum are achieved for the channel conductivity and the radii characteristic of lightning.

**Figure 8:**
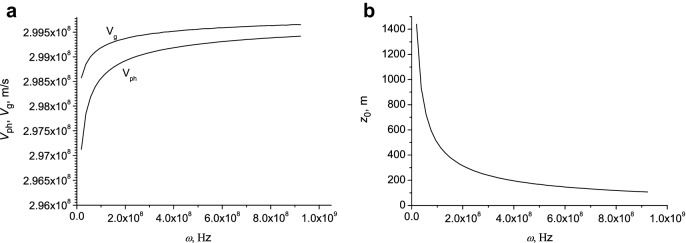
Phase and group velocity (**a**), and attenuation length (**b**) as function of frequency.

In Fig. 8a the phase and group velocities are presented as a function of surface wave frequency. It is seen that *V*_g_ > *V*_ph_. In Fig. 8b the dependence of the attenuation length on the frequency is shown.

The group velocity $V_{g} = \frac{d\omega }{{d\beta^{\prime}}}$ can be expressed as $V_{g} = V_{ph} - \lambda \left( {\frac{{dV_{ph} }}{d\lambda }} \right)$. Since $\frac{{dV_{ph} }}{d\lambda}<0$, then *V*_g_ > *V*_ph_. This indicates that abnormal dispersion occurs at these frequencies. The group velocity determines the velocity of propagation of the amplitude of the envelope of a wave packet formed as a result of interference (beats) of waves with close frequencies. Note that in a non-dispersive medium, the group velocity of the guided modes is less than the phase velocity^43^.

### Photon frequency

**Figure 9:**
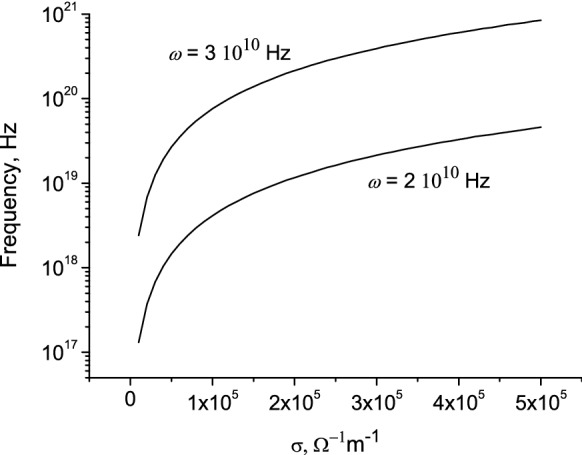
Photon frequency as function of conductivity. $r_{0}$ = 0.01 m.

In Fig. 9 the photon frequency $\omega_{c}$ as function of the conductivity is presented for different frequencies of the surface wave.

**Figure 10:**
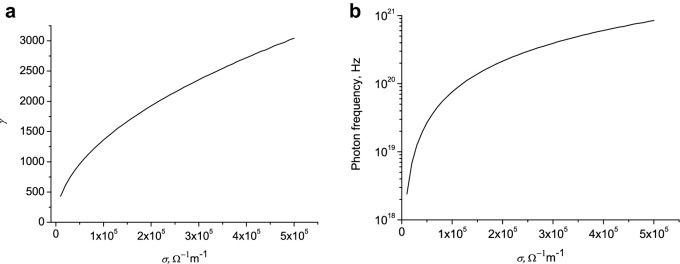
The Lorentz factor (**a**) and photon frequency (**b**) as function of conductivity. $\omega = 2\pi f = 3 \cdot 10^{10}$ Hz, $r_{0}$ = 0.01 m.

In Fig. 10a the values $\gamma = \left( {1 - \frac{{v^{2} }}{{c^{2} }}} \right)^{{ - \frac{1}{2}}}$ as a function of the conductivity are presented. The values of γ for a given conductivity and the radius of the lightning channel are determined from the solution of Eq. (1). In Fig. 10b the photon frequency as a function of the conductivity is shown.

Thus, the spectrum of synchrotron radiation of lightning covers almost the entire scale of electromagnetic waves—from the radio frequency to the X-rays and gamma rays.

**Figure 11:**
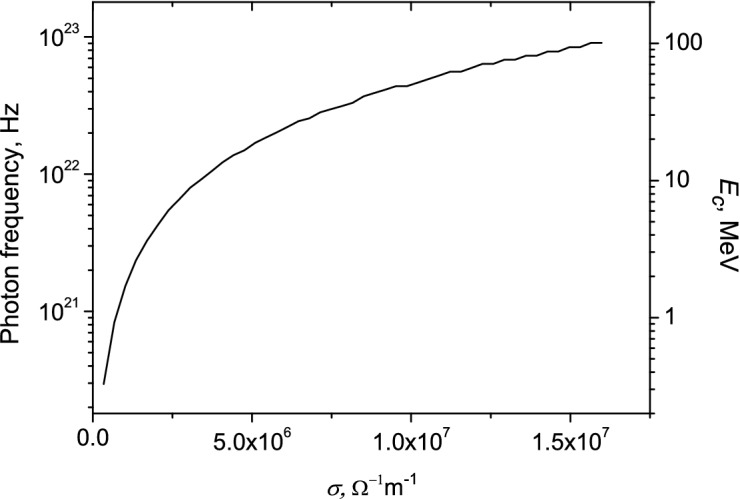
The photon frequency and energy as function of conductivity. $\omega = 2\pi f = 3.1 \cdot 10^{10}$ Hz, $r_{0}$ = 0.01 m.

In Fig. 11 the photon frequencies $\omega_{c}$ and photon energies $E_{c} = \omega_{c} \hbar$ corresponding to these frequencies as a function of the conductivity are shown.

In Table 1 the attenuation lengths, Lorentz factors, photon frequencies and energies are presented for different values of the channel conductivity and surface wave frequency.

**Table 1:** Relationships between lightning and X-ray and gamma-ray radiation parameters. $r_{0}$ = 0.01 m, $f = \frac{\omega }{2\pi }$.

*σ*, Ω^−1^ m^−1^	*f*, Hz	*Z*_0_, m	γ	$\omega_{ph}$, Hz	*E*_*ph*_, eV
10^4^	10^8^	175	17	1.48 × 10^14^	0.1
	10^9^	27.2	27.7	0.64 × 10^15^	0.42
	5 × 10^9^	4.2	441	2.57 × 10^18^	1690
10^5^	10^8^	556	31.7	0.96 × 10^15^	0.63
	10^9^	85.5	52.8	4.4 × 10^15^	2.9
	5 × 10^9^	13.3	1393	8.1 × 10^19^	53350
10^6^	10^8^	1667	59	6.16 × 10^15^	4.05
	10^9^	267	100	3 × 10^16^	19.7
	5 × 10^9^	42	4389	2.54 × 10^21^	1670000

### Photon number

The number of photons with frequencies $\omega_{max} \approx \omega_{0} \gamma^{3}$ radiated for the time $\delta t \approx \frac{1}{{\omega_{0} }}$ of the electron's revolution around the circle is given by

8 $$N_{ph} \approx \frac{W}{{\omega_{0 } \hbar \omega_{max} }} \approx \frac{{e^{2} }}{{\hbar {\text{c}}\varepsilon_{0} }}\gamma \approx \frac{{2.56{ \times }10^{ - 38} \gamma }}{{1.05{ \times }10^{ - 34} { \times }3{ \times }10^{8} { \times }8.85{ \times }10^{ - 12} }} \approx 10^{ - 1} \gamma .$$

This number of photons corresponds to the radiation over time $\delta t \sim 10^{ - 10}$ s. For a time of 1 μs, the number of photons is equal to $N_{ph} \sim 10^{3} \gamma$.

For single charges, the radiation is weak. The situation changes when we consider bunches of particles. Indeed, the polarization of the plasma occurs in a certain extended region, so as a result, the amplitude of the polarization radiation will be determined by the total dipole moment of the bunch. In this case, a bunch of polarized plasma will radiate like point particles with a charge and multipole moments corresponding to the entire bunch.

The power of synchrotron radiation consists of coherent and incoherent parts^36^:

$$W\left( m \right) = W^{incoh} \left( m \right) + W^{coh} \left( m \right).$$

When radiation is incoherent, the total power is given by^36^:

$$W_{N} \left( m \right) = N_{e} \cdot W\left( m \right),$$

where *N*_*e*_ is the number of electrons in a polarized plasma bunch.

The radiation in the high-frequency range is incoherent and proportional to the number of electrons^36^: $W_{N} = N_{e} \cdot W$. The estimated total number of photons allows us to explain how a large number of gamma rays are produced. Note that the typical brightness of a TGF observed from space is within an order of magnitude of 10^17^—10^19^ gamma rays^9,38^. This value corresponds to the number of electrons $N_{e} \approx 10^{12 \div 13}$ for $\gamma = 10^{3}$.

The current density in Maxwell equation for the electric field acquires an additional term:

9 $$\overline{j} = \sigma \overline{E} + \frac{{\partial \overline{P}}}{\partial t} = \sigma \overline{E} + \varepsilon_{0} \left( {\varepsilon - 1} \right)\frac{{\partial \overline{E}}}{\partial t},$$

where $\overline{j}_{cond} = \sigma \overline{E}$ is the conduction current and $\overline{j}_{pol} = \frac{{\partial \overline{P}}}{\partial t}$ is the polarization current.

Number of electrons in a polarized plasma bunch is determined by the polarization current $i_{pol}$:

$$N_{e} \simeq \frac{Q}{e} \simeq \frac{{i_{pol} \cdot \Delta t}}{e},$$

where $$\Delta t \sim \tau_{f} \sim \frac{1}{\omega } \sim 10^{ - 10}$$ s.

For the number of electrons $N_{e} \approx 10^{12 \div 13}$ we obtain that the polarization current will be of the order of

10 $$i_{pol} \simeq \frac{{N_{e} \cdot e}}{\Delta t} \sim \frac{{10^{12 \div 13} \cdot 1.6{ \times }10^{ - 19} }}{{10^{ - 10} }} \sim 10^{3 \div 4} \;{\text{A}}{.}$$

Note that the magnitude of the conduction current amplitude during the return stroke is about 30 -100 kA^10,25^.

The radiation power increases dramatically in the case of coherent bunch of electrons grouped at distances less than the wavelength of the emitted wave. The coherent radiation can be observed in low-frequency range, i.e., in the radio-frequency one. It is known that the radio-frequency pulses of pulsars are emitted by a coherent synchrotron mechanism^44^. The radiation power increases dramatically in the case of coherent bunch of electrons grouped at the distances less than the wavelength of the emitted wave. In this case the radiation power is given by $W^{tot} = N_{e}^{2} \cdot W$.

High-intensity microwave radiation of a lightning discharge was observed in^25,45,46^. The extreme brightness of the radiation observed from fast radio bursts indicates that it is generated in the process of coherent radiation. It was shown in^45^ that the microwave radiation from lightning is a sequence of individual pulses, and the radiation spectrum differs from the spectrum in the long-wave range. This indicates that in the decimeter range, the mechanism of generating electromagnetic waves differs from the usual dipole radiation of the lightning current. Recently it was shown that each leader step emits a burst of multiple discrete VHF pulses^15^.

### Transition radiation

Another mechanism for producing microwave, X-ray and gamma-ray during the propagation of a surface wave along the discharge channel is the transition radiation^47^. Transition radiation occurs when polarization distribution (electric dipoles) moves at a speed exceeding the phase speed of light in the medium. However, the power of transition radiation is much lower than that of synchrotron radiation. The power increases greatly with coherent transient radiation.

The total energy radiated by an electron is given by^47^:

11 $$P = \frac{{e^{2} \omega_{p} }}{{3c\varepsilon_{0} }}\gamma ,\;\omega_{p}^{2} = \frac{{ne^{2} }}{{m\varepsilon_{0} }}.$$

The number of photons is determined by

12 $$N_{ph} = \frac{P}{{\hbar \omega_{ph} }} = \frac{{e^{2} \omega_{p} }}{{3c\varepsilon_{0} \hbar \omega_{ph} }}\gamma .$$

This number is much smaller than that of synchrotron radiation. However, the number of photons increases significantly with coherent transient radiation, i.e. for the radio frequency range.

## References

[1] Wilson CTR (1925). The acceleration of β-particles in strong electric fields such as those of thunderclouds. Proc. Cambridge Phil. Soc..

[2] Enoto T (2017). Photonuclear reactions triggered by lightning discharge. Nature.

[3] Fishman GJ (1994). Discovery of intense gamma-ray flashes of atmospheric origin. Science.

[4] Dwyer JR (2012). Observation of a gamma-ray flash at ground level in association with a cloud-to-ground lightning return stroke. J. Geophys. Res..

[5] Wada, Y., et al. Downward Terrestrial Gamma-Ray Flash Observed in a Winter Thunderstorm. *Phys. Rev. Lett.* **123**, 061103 (2019). 10.1103/PhysRevLett.123.061103 31491171

[6] Schaal MM (2012). Spatial and energy distributions of X-ray emissions from leaders in natural and rocket triggered lightning. J. Geophys. Res..

[7] Kochkin PO, van Deursen APJ, Ebert U (2015). Experimental study on hard x-rays emitted from metre-scale negative discharges in air. J. Phys. D Appl. Phys..

[8] Gurevich AV, Milikh GM, Roussel-Dupre R (1992). Runaway electron mechanism of air breakdown and preconditioning during a thunderstorm. Phys. Lett. A.

[9] Dwyer JR, Smith DM (2005). A comparison between Monte Carlo simulations of runaway breakdown and terrestrial gamma-ray flash observations. Geophys. Res. Lett..

[10] Dwyer JR, Uman MA (2014). The physics of lightning. Phys. Rep..

[11] Xu W, Celestin S, Pasko VP (2014). Modeling of X-ray emissions produced by stepping lightning leaders. Geophys. Res. Lett..

[12] Babich LP, Bochkov EI, Kutsyk IM, Neubert T, Chanrion O (2015). A model for electric field enhancement in lightning leader tips to levels allowing X-ray and ray emissions. J. Geophys. Res. Space Phys..

[13] Tavani M (2011). Terrestrial gamma-ray flashes as powerful particle accelerators. Phys. Rev. Lett..

[14] Pleshinger DJ (2019). Gamma ray flashes produced by lightning observed at ground level by TETRA-II. J. Geophys. Res. Space Phys..

[15] Hare BM (2020). Radio emission reveals inner meter-scale structure of negative lightning leader steps. Phys. Rev. Lett..

[16] Neubert T (2020). A terrestrial gamma-ray flash and ionospheric ultraviolet emissions powered by lightning. Science.

[17] Alnussirat ST, Christian HJ, Fishman GJ, Burchfield J, Cherry ML (2019). Simultaneous space-based observations of terrestrial gamma-ray flashes and lightning optical emissions: Investigation of the terrestrial gamma-ray flash production mechanisms. Phys. Rev. D.

[18] Heumesser M (2021). Spectral observations of optical emissions associated with Terrestrial Gamma-Ray Flashes. Geophys. Res. Lett..

[19] Sommerfeld A (1899). Uber die Fortpflanzung elektrodynamischer Wellen langs eines Drahtes. Ann. Phys. Chem..

[20] Uman MA (1964). The diameter of lightning. J. Geophys. Res..

[21] Orville RE, Helsdon JH, Evans WH (1974). Quantitative analysis of a lightning return stroke for diameter and luminosity changes as a function of space and time. J. Geophys. Res..

[22] Cen J (2018). Electron density measurement of a lightning stepped leader by oxygen spectral lines. AIP Adv.

[23] Cen J, Yuan P, Xue S, Wang X (2015). Resistance and internal electric field in cloud-to-ground lightning channel. Appl. Phys. Lett..

[24] Petrov NI, Waters RT (1995). Determination of the striking distance of lightning to earthed structures. Proc. R. Soc. A.

[25] Petrov NI, Waters RT (2021). Lightning to earthed structures: striking distance variation with stroke polarity, structure geometry and altitude based on a theoretical approach. J. Electrostat..

[26] Krider EP, Leteinturier C, Willet JC (1996). Submicrosecond fields radiated during the onset of first return strokes in cloud-to-ground lightning. J. Geophys. Res..

[27] Petrov NI, Avanskii VR, Bombenkova NV (1994). Measurement of the electric field in the streamer zone and in the sheath of the channel in a leader discharge. Tech. Phys..

[28] Loeb LB (1965). Ionizing waves of potential gradient. Science.

[29] Vasilyak LM, Kostyuchenko SV, Kudryavtsev NN, Filyugin IV (1994). Fast ionisation waves under electrical breakdown conditions. Phys. Usp..

[30] Liang C, Carlson B, Lehtinen N, Cohen M, Marshall RA, Inan U (2014). Differing current and optical return stroke speeds in lightning. Geophys. Res. Lett..

[31] Carvalho FL, Jordan DM, Uman MA, Ngin T, Gamerota WR, Pilkey JT (2014). Simultaneously measured lightning return stroke channel-base current and luminosity. Geophys. Res. Lett..

[32] Thottappillil R, Schoene J, Uman MA (2001). Return stroke transmission line model for stroke speed near and equal that of light. Geophys Res. Lett..

[33] Petrov NI, Petrova GN (1999). Physical mechanisms for the development of lightning discharges between a thundercloud and the ionosphere. Tech. Phys..

[34] Schott GA (1907). Electromagnetic radiation and the mechanical reactions arising from it. Ann. der Phys..

[35] Sokolov AA, Ternov IM (1957). On polarization effects in the radiation of an accelerated electron. Sov. Phys. JETP.

[36] Ternov IM (1995). Synchrotron radiation. Phys. Usp..

[37] Ginzburg VL, Syrovatskii SI (1966). Cosmic magnetic bremsstrahlung (synchrotron) radiation. Sov. Phys. Usp..

[38] Mailyan BG (2016). The spectroscopy of individual terrestrial gamma-ray flashes: constraining the source properties. J. Geophys. Res. Space Phys..

[39] Lyu F, Cummer SA, McTague L (2015). Insights into high peak current in-cloud lightning events during thunderstorms. Geophys. Res. Lett..

[40] Belz JW (2020). Observations of the origin of downward terrestrial gamma-ray flashes. J. Geophys. Res. Atmos..

[41] Saba MMF (2019). High-speed video observation of a dart leader producing X-rays. J. Geophys. Res. Space Phys..

[42] Mailyan BG (2020). Gamma-ray and radio-frequency radiation from thunderstorms observed from space and ground. Sci. Rep..

[43] Petrov NI (2019). Speed of structured light pulses in free space. Sci. Rep..

[44] Michel FC (1982). Intense coherent submillimeter radiation in electron storage rings. Phys. Rev. Lett..

[45] Kosarev EL, Zatsepin VG, Mitrofanov AV (1970). Ultrahigh frequency radiation from lightnings. J. Geophys. Res..

[46] Petersen D, Beasley W (2014). Microwave radio emissions of negative cloud-to-ground lightning flashes. Atmos. Res..

[47] Ginzburg VL (1996). Radiation by uniformly moving sources (Vavilov—Cherenkov effect, transition radiation, and other phenomena). Phys. Usp..

